# Intersectionality of cultural norms and sexual behaviours: a qualitative study of young Black male students at a university in KwaZulu-Natal, South Africa

**DOI:** 10.1186/s12978-020-01041-3

**Published:** 2020-11-24

**Authors:** Sinakekelwe Khumalo, Myra Taylor, Tawanda Makusha, Musawenkosi Mabaso

**Affiliations:** 1 Discipline of Public Health, School of Nursing and Public Health Medicine, University of KwaZulu-Natal, Durban, South Africa; 2 Human and Social Capabilities Research Programme, Human Sciences Research Council, Durban, South Africa

**Keywords:** Culture, Norms, Sexual behaviour, Sexual risks, Male students, University, South Africa

## Abstract

**Background:**

Sexual risk behaviours that occur among young men are based on dominant notions and practices that prevail in cultural contexts. As such, understanding the intersection of cultural norms and sexual risk behaviours among young men is very important.

**Methods:**

The study used a qualitative design and conducted four focus group discussions with 36 male students who were purposively selected from different levels of study at the University of KwaZulu-Natal. Data were analysed through line-by-line coding, and grouped into emerging themes and sub-themes facilitated by the use of Atlas.ti.

**Result:**

The findings emphasize that socialisation agents such as the family, peers and community play an important role in prescribing acceptable and unacceptable sexual behaviour of young men. Some of the young men seemed to adhere to prescribed gender norms of what it means to be a man while some rejected them for alternative versions of being a man. In the context of the university environment, these findings reveal that male students cannot make informed decisions regarding condom use when they are intoxicated, and thus expose themselves to sexually transmitted infections and other risks.

**Conclusion:**

University sexual risk reduction programs should be developed considering the specific cultural context, using strategies that empower young men to challenge the widely accepted cultural norms that may predispose them to sexual risks.

**Plain English summary:**

Sexual behaviours and cultural norms are interconnected, it is through culture that people learn how to behave and understand the world around them. In many cultural contexts, young men are taught from a very young age how to behave based on dominant notions of what it means to be a man in that particular context. As such, in some cultural context sexual risk-taking such as having multiple sexual partners and unprotected sex are perceived as normal behaviour for men. Some young men embrace such normalised sexual behaviours which often has negative implications on their future. This study explored the influence of cultural norms on the sexual behaviour of young men. This qualitative study was conducted at the University of KwaZulu-Natal. Four focus group discussions were conducted among first-year students to postgraduate students who were between the ages of 18 to 30 years. Our findings revealed that there other influences on the sexual behaviours of the young men, which included family, community and peers. It also emerged that gender norms regarding what it means to be a man still prevailed which some of the young men in the study adhered to, notably such notions seemed to be rejected by some of them. The university setting appeared to be space where a lot of sexual risk-taking took place, which potentially exposed the young men in the study to many sexual risks. In conclusion, targeted programs for the university setting should aim to challenge gender norms that expose young men to sexual risks.

## Background

The intersection between culture and sexual behaviours is very much contested. Studies show that particular cultural norms influence certain sexual behaviours [1–5]. Other scholars disagree on what these cultural norms are, how they sustain sexual behaviour and how they can be changed [6, 7]. However, whilst there is a general agreement that norms influence behaviour, there are several different theories on exactly when and how they do so [8, 9]. Previous research has found that cultural norms that relate to what it means to be a man have a significant influence on young men’s sexual behaviours [10]. As such it becomes important to understand the context of cultures and traditions when talking about sexual activities [3]. Sexual activities in many African settings is perceived to be an important expression of men’s masculinity as sex is viewed as an activity of fun and fame [5]. Men continue to be praised for their sexual prowess and prevailing beliefs that men’s sexual desires are uncontrollable while [11] which thus perpetuate norms such as multiple sexual partnerships among men. According to Morrell, Jewkeys and Lindegger [12] norms related to masculinity are culturally informed and young boys are expected to conform to these set ideals. Researchers also argue that there may be other factors besides cultural norms influencing sexual behaviours, such as individual will, peer pressure, gender and age [13–15].

In this paper, we use the definition put forward by recent work in social psychology, namely that norms are: (a) beliefs about what others in a given group do (that is, what is typical in the group); and, (b) beliefs about what others in a given group approve and disapprove of (that is, what is appropriate in the group) [6, 7, 16–18]. Cultural practices can be powerful drivers of behaviour because these are standards people live by [19]. They are shared expectations and rules that guide the behaviour of people within particular social groups [20]. There are clear expectations that young men have to adhere to as they are constantly judged and assessed as to whether they live up to these expectations [21]. The context becomes important in prescribing and endorsing certain norms and behaviours. Consequently, young men construct their sexual behaviours based on such dominant notions and practices that prevail in their cultural context. According to [22] young men who were socialised to adopt traditional masculine norms change their masculine ideology when they go to university as they are become exposed to different and liberal cultural practices which exist within higher education institutions. There is a large body of research indicating how socialisations produce different forms of masculinities [23–26]. Barker and Ricardo [21] stated that among young men in Africa versions of masculinities are multiple, with the conflicting understanding of what it means to be a man.

The way we were raised influences our behaviours whether negative or positive. As such, it becomes more important to understand the context where behaviours are constructed and shaped. The explanation of male students’ sexual behaviours can be determined through understanding the meaning and influence that they attached to the cultural norms related to sexual behaviours. Thus, an understanding of the context within which individuals become socialized enables a better understanding of why individuals act and behave in certain ways [27–30]. For individuals to assume their social roles, socialization into particular cultural practices and beliefs needs to take place [31, 32]. As socialisation is reliant on institutions such as the family, community, peers, schools and churches [28, 33]. Individuals thus become integrated into the cultural norms of society through socialization [34]. The norms are positioned by communities and other social agents to influence certain behaviours.

In this paper, we explore the intersectionality of cultural norms related to sexual behaviours among Black male students in Black male students at the University of KwaZulu-Natal, South Africa.

## Methods

A qualitative research methodology was used to explore the cultural norms and sexual behaviours among young men attending an institution of higher learning at the University of KwaZulu-Natal (UKZN) in South Africa. The qualitative research approach was found useful for this study as it stresses the way people interpret and make sense of their lived experiences [35, 36]. Qualitative methods enabled the study to yield rich data in exploring the influence of cultural norms on the sexual behaviours of young men.

### Study site and participants

The study was conducted at (UKZN). The university comprises of five campuses namely Howard College Campus, Westville Campus, Pietermaritzburg Campus, Edgewood Campus, and the Nelson Mandela School of Medicine. The rationale for selecting UKZN as a study site was because of its attraction for a large number of students from diverse social and cultural backgrounds across South Africa and other parts of sub-Saharan Africa. This cross-cultural diffusion made UKZN a rich study site. The study was conducted at Howard College Campus because it is the largest and home to most of the faculties at the institution. This study was conducted in September 2018 to November 2018.

### Study procedure

The study employed a purposive sampling technique to recruit Black male students who were between the ages of 18–30 years. The inclusion criteria were that all participants had to be Black (regardless of ethnicity), identify themselves as male, and had to be studying at the university (both undergraduate and postgraduate students were included). The participants were recruited from the four Colleges at the Campus, namely College of Agriculture, Engineering and Sciences, College of Health Sciences, College of Humanities and College of Law and Management studies. To recruit the participants, the researcher (SK) designed posters and flyers with the study description and contact details of the researcher. With permission granted by the university, the researcher distributed these posters and flyers across the campus. A total number of 16 male students initially responded, and snowball sampling was used to recruit additional participants.

### Instruments

Focus group discussions (FGDs) were used to collect the data. A total of four FGDs were conducted with a total of 36 male participants who were between the ages of 18–30 (see Table 1). Majority of our participant came from rural areas with a few coming from urban areas. This method was found to be useful as it enabled the researcher to capture dynamic perceptions, understanding, beliefs and attitudes of young men through group interactions [37, 38]. This method also allowed the researcher to understand how young men construct their sexual behaviours and how dominant cultural norms that exist in their families, community and among peers influences their behaviour in the space of the university.

**Table 1:** Description of focus group

Focus group discussion (FGD)	Level of study	Ages	Ethnicity	Sample size (N = 36)
FGD 1	First-year students	18–21	Zulu and Xhosa	10
FGD 2	Second-year students	19–23	Zulu and Xhosa	8
FGD 3	Third-year students	23–29	Zulu, Tswana, Sotho, and Xhosa	10
FGD 4	Post-graduate students (honours, masters and PhD)	24–30	Zulu, Xhosa, Venda and Sotho	8

The focus group discussions covered a range of topics related to dominant sexual practices and cultural norms. Some questions asked about the general norms that existed among the communities where the young men come from, norms that existed within their families and among peers. We asked questions specifics questions such as (i), In your community, what are the sexual behaviours that are acceptable for men, (ii) What is the acceptable age for initiation of sexual intercourse, (iii) What are your views on multiple sexual partnerships and (iv), Are there any traditional practices in your culture that teach and educate men about sex and sexual behaviours.

### Data analysis

All FGDs were conducted in English, audio-recorded and transcribed. Data were analysed thematically, guided by a thematic analysis framework [39]. The first author read and re-read all the transcripts to familiarize herself and get a better sense of the narratives of the participants. After familiarisation with transcripts, codes were generated as guided by the study questions and produced through the use of *Atlas. ti 8*. Further categorisation of codes into emerging themes was conducted by the first, second and third authors focusing on the connections between the emerging themes until consensus was reached.

### Ethical consideration

Ethical approval was obtained from UKZN’s Humanities and Social Sciences Research Ethics Committee (HSSREC) (Protocol number: HSS/0255/018D). The study information sheet was read to all participants and they were also given study information sheets containing details about the study. All participants were requested to sign an informed consent form before they were enrolled in the study. The study information sheet and consent form explained the maintenance of confidentiality for any information provided during the FGDs for the purposes of this study. Participants were informed that their real names would not be used at any point in the study and only pseudonyms would be used where necessary. Participants were also reminded of their rights to withdraw from the study at any time, in case they felt uncomfortable or if they felt that the study was an inconvenience to them and that such withdrawal from the study would not have any negative repercussions for themselves or their studies.

## Findings

Four pervasive themes emerged from the young men’s narratives on the influences of cultural norms on their sexual behaviours. These comprised (1) social background and upbringing, (2) cultural norms and sexual history, (3) gender norms and sexual scripting, and (4) university culture and sexual risk behaviour.

### Social background and upbringing

The family, peer groups and community setting are important social agents in the upbringing of an individual. Across the focus groups, participants narrated that their environment which constituted their family, peers and community played an important role in the construction of their sexual behaviours. Our data indicated that the sexual norms that prevailed in the families, communities and peer groups of the young men shaped their sexual behaviours and attitudes. One participant stated that:

*“In the community, I was raised in, the majority of the men had multiple sexual partners…so having as many partners as possible was normalised, some men were in polygamous relationships … but I also had to deal with my family as well. The family values that they instilled in about sexual behaviour were different from the ones I learned from the community”.* (FGD 3_3^rd^ year student).

In support of the above statement, another participant recounted:

*“Outside there [in] the community, people would praise you. But at home bringing different girls would never be tolerated”* (FDG 2_2^nd^ year students). This statement shows the different and competing norms that are prevalent in the community setting as opposed to those that are within the family. One other participant in the same group noted:

*“In my family, multiple sexual partners were frowned upon… it [was] just immoral and not the manly thing to do because of the way they look[ed] at it … you know they are very traditional in terms of family values and so on. So you have to have one partner [which] you are committed to so that there could have a real potential so that you could start a family with that person, carry the family name forward…every man in the family got married at a very young age, so you knew that you could not get involved with many women.”* (FDG 2_2^nd^ year student).

This highlights the important role played by the family in the construction of sexual behaviour as it provides role models and set standards of appropriate sexual conduct of young men. Apart from the family, there was general agreement among the participants that peers played a significant influence on their sexual behaviours. One participant stated*: “A guy needs to have many girlfriends and makes sure he sleeps with them”* (FGD 2_ 2^nd^ year student).

This was supported by one participant in a focus group with Postgraduate students who narrated:

“*All of my friends were already drumming [having sex] honestly, so I asked them how to do it [sex]. It was trial and error for me and my friends in the beginning but after some time, we were teaching others and moving from one girl to next*” (FGD 4_Postgraduate student).

This is because for many young men sexual experience is perceived as a social and cultural rite of passage through which every young man has to pass.

Interestingly, a few participants who came from religious backgrounds, considered their religious beliefs very influential. The participants were of the view that their religious upbringing provided them with a set of moral standards that did not sanction premarital sex as it was considered a sin. The following account captures one of the participant’s views (FGD 3_3^rd^ year student):

“*I’m from a strong Christian family so what [I] was taught [was that] sex before marriage is a sin. So that’s why even now as a young man I’m abstaining from it until I get married.*”

As the participant recounted this, majority of the participants were shocked and some mocked him. This occurred with all the participants who admitted to being virgins, across all the focus group discussions. Virginity was perceived as shameful and as something that each young man needed to get rid of, to avoid humiliation. Hence another participant in the same group of second-year students recounted:

*“Like some of my brothers in this room I come from a very strong Christian family my grandfather is a pastor, my father is also a pastor as well, so I did not follow some of the teachings. Even though I went to a Christian school where we used to stand in a line as young men and we would be proud to be virgins. When I left school I carr[ied] those sexual norms to boarding school and I was a laughing stock. You could not be 16 years old and a virgin so it was not a comfortable thing. As proud as I was then, I made sure that I lost my virginity*” (FGD 3_3^rd^ year student).

The narrative highlights that even with a strong religious background, for many young men the pressure to live up to dominant prescribed sexual norms was overpowering. As such, personal beliefs, behaviours and attitudes were not consistent, as they were constantly being influenced by other norms that existed in their communities and among their peers.

### Cultural norms and sexual history

The young men in the study discussed in detail their own unique sexual life experiences, and this discussion enabled some young men to reflect on their subjective sexual histories. For some of the participants, their first sexual experience seemed to have shaped their understanding and the meaning that they attached to their sexual practices to date. Some young men reported that their first experience of sex was often accompanied by pressure that was exerted on them by external forces such as peers and by prevailing social norms in their communities. One participant noted.

“*I grew up in a community where having sex and a girlfriend as a young man was expected…There is a stigma attached to being a virgin. As a man where I come from, all of my friends had already had sex, I knew I had to have sex as well”.* (FGD 4_Postgraduate students).

This suggested that for some of the participants their first sexual experience was motivated by curiosity and modelling of the male figures in their lives. This placed in question their readiness and understanding of what they were doing.

This was further emphasised when a majority of the young men reported their sexual debut by the age of 15, for some even at the age of 11, with no condom use reported at their first sexual encounter. The following extract depicts a participant’s experience who recounted his first sexual encounter (FGD 4_Postgraduate students):

“* I started when I got this girl who already had sex, so I didn’t have a problem it was easy for me because she already had experience with other guys, we did it* [had sex] *in the bush because we could not do it at home. I was very quick, I remember I did not even use protection at that time. I did not even know about condoms and stuff. Even though I had not had sex before, I did watch porn and I also learned from other guys and older brothers on what to do, like the withdrawal method…but I could not do it, it was too nice”.*

Given the prevalence of HIV among the age group of 15–24 in South Africa, the above narration highlights troubling issues. Firstly, the lack of knowledge regarding safer sexual practices. Secondly, the sexual risks involved in such behaviours, which for this participant like many other participants, were during their first sexual experience. Thirdly, getting sexually intimate without the use of protection with a woman who previously had other sexual partners, opened up risks for onset exposure to sexually transmitted infections and lastly, the mentioning of the withdrawal method suggested that there was more attention on preventing pregnancy, rather than HIV and other sexually transmitted infections.

The beliefs, knowledge and values that are attached to sexual behaviour by an individual are based on the dominant sexual scripts that are prevalent in a particular cultural context. This was evident among young men from traditionally circumcising communities, as they stated that they were taught about responsibility which included safer sexual practices during initiation. However, some misconceptions were prevalent in such contexts. Participants reported that many people in their communities believed that a man who was circumcised could not be infected with HIV and other sexually transmitted infections. Moreover, such normalised beliefs around circumcision also promoted non-condom use for them and many other people in their communities. This is depicted in the following:

*“Back at home we do have conversations about safe sex practices, they say we should get circumcised so that we can have sex without the use of a condom. The general belief from a lot of people back at home is that a man who is circumcised has small chances of getting infected with HIV “.* (FGD 1_1^st^ year student).

This revealed the extent to which the social context shapes the perceptions and attitudes of young men in how they perceive sexual risk. With the use of condoms, the narrative above highlights how prevailing norms, understanding and knowledge regarding condom use, influenced how, when and whether these young men used condoms. Moreover, the narratives also indicated the lack of knowledge about circumcision and the continued use of protection post circumcision in such settings. In many African families, discussion about any sexually related topics is forbidden. However, for some of the participants when they recounted their sexual history they indicated that, when they started engaging in sexual activities, their fathers played an important role in educating them about the use of condoms.

*“I remember when I started having sex, I was around the age of 15 or 16. My father told me that he suspects that I might be having sex or that I will be having it at some point, so he told me to be safe and always use a condom every time I had sex with a woman”.* (FGD 4_Postgraduate student). This was considered surprising to hear by some of the young men in the group, who openly stated that talking about sex with their parents was seen as culturally inappropriate. The common belief in their communities according to the young men was that sex talk was reserved for adults only.

### Gender norms and sexual scripting

In the African culture, the understanding of gender tends to be viewed according to specific norms and expectations, into which young men and young women are socialised at a very early age. The expectations are structured and constructed by culture and society, and they shape the sexual behaviours of both men and women. They further influence how young men and young women interact and navigate in sexual relationships. Our data illustrate the extent to which the constructions of gender norms have impacted on the sexual behaviours of the young men in the study. The recurring description of the gender norms that some young men in the study were taught at a young age was that a man is a “leader” or “head”. Such socialisation cemented notion and understanding that made many of the young men to believe that they always had to control women and their sexual interactions with women. A young man in his first year of study narrated:

“*The way society sees a man is through his actions and in terms of doing all the things that are expected of a man to do in terms of societal norms*”. (FGD 1_1^st^ year student).

In their narrations in the group discussions, it became clear that for some of these young men, their understanding of gender roles was linked to the performance of observable behaviours that were dominant in their cultural context. This, in turn, informed their behaviour and the need to adhere to socially constructed expectations by all means. On the other hand, there appeared to be individuals who felt the need not to conform to the expected gender roles and adopt alternative versions of “being a man”. For some of these young men, their understanding of gender norms was distinctly different. They did not want to prescribe to gendered norms and cultural ideals, where emotions are perceived as weakness, and violence and aggression were viewed as a strength. They aspired to express their manliness in alternative ways. This was particularly evident among participants who were part of the Honours’ and Masters’ focus group discussion.

“*There are things that were taught when I was growing up about what it means to be a man, some of these things did not make sense to me. For example, having multiple sexual partners was portrayed as the norm. I have adopted a different way of being a man, which is totally different from the normalised norm”*. (FGD 4_Postgraduate student).

However, some of the young men did not reject the dominant gender norms. For example, this was evident when one participant in a group discussion stated that “*A girl who has many sexual partners is regarded as a ‘bitch’*. He further explained: “*They* [referring to people in his community] *do not see her in a good way…In most cases, the guys will just go to her and sleep* [have sex] *with her and without commitment. Our elders have warned and advised us against such girls as they are considered not ‘wife material’* (FGD 1_1^st^ year students)*.*

The reason behind such notions might be because culturally, women are expected to remain virgins until marriage, whilst expecting men to be knowledgeable about sex. There also appeared to be a dichotomy in the teaching received by young people.

“*When girls are taught that they should behave and not have sex until marriage, whilst we are encouraged to have sex…it becomes tricky for me, who must we have sex with? They have to teach us good behaviour as”.* (FGD 4_Postgraduate student).

In many cultural settings in Africa, more emphasis regarding appropriate behaviour has always targeted women, while giving little focus on appropriate behaviours among young men. Reflecting on this was one of the participants in (FGD 2_2^nd^ year students) who recounted:

*“A man with many sexual partners is a real man! It means his penis is working for real, he is a real bull. I cannot say the same thing for a woman, a woman who does the same is a slut* [the majority of participants were in agreement with this]. *A woman needs to control herself, like (she) has to control her sexual feelings.”*

The above statement points to the symbolic meaning that is attached to sexual prowess and sexual virility among these young men. This is quite evident in many cultural settings where men who cannot function sexually are often emasculated [40, 41]. Hence, hypersexual behaviours are used as a demonstration of manhood and to attain respect and power from other men.

### University culture and sexual risk behaviour

Research has shown that entering university is one of the important steps in one’s life course and is associated with many changes in lifestyle such as experimenting with alcohol and drugs because of newfound independence [42]. This has critical implications for sexual behaviour especially for young people in the university space. Similarly, Our findings suggested that alcohol seems to influence young men’s sexual behaviour. There was a general agreement among the participants that they were unlikely to use a condom when they have been drinking. This is highlighted by the following statement:

*“When you are intoxicated it makes it difficult to think, alcohol takes away rationality. When you put alcohol on the table, obviously you are not going to use a condom…You will be in a rush… no one thinks when they are in a rush”.* (FGD 2_2^nd^ year student).

The university became a space for these young men to sexually explore without getting into committed relationships. The participants spoke about unplanned sexual encounters as widely accepted in the university setting, thus showing the normalization of risky sexual behaviours. Participants stated that such unplanned sexual encounters were more common when they were intoxicated. In the same vein, across all the focus group discussions, a majority of the participants reported on unsafe sexual practices such as non-condom use and the use of lubrication during unprotected sex to prevent friction and tear of the vaginal area, because of the belief that they would not get infected with HIV since the vaginal area would be well lubricated. These findings are troubling especially because such practices put these young men at risk of contracting HIV and other sexually transmitted infections. Third-year students recounted the following:

*“It is a norm here in university to randomly sleep around with no strings attached, I have friends that would tell you that they have slept with a random girl on campus, and they would even point you to some of the girls when we are in lecture rooms. It has become normal….It is no longer scary”.* (FGD 3_3^rd^ year student).

Particularly troubling about the above statement is that the university space has been represented as an enabling environment for risk-taking, where momentary pleasure outweighs the consequences. More disturbing was that the risk-taking reported commonly involved the consumption of alcohol, thus perpetuating risky sexual behaviours such as unprotected sex and inconsistent condom use among young people. Our data also showed the presence of transactional sex among this group of young men where alcohol was exchanged for sexual favours from women. A postgraduate student explained: *“When we go to the club I make a girl happy by buying her booze* [alcohol], *I will be happy because she will make me happy by sleeping with me*”. (FGD 4_Postgraduate student). In their narration, it was evident that this expectation from women was implicit, but it was a common expectation to which according to the participants, women adhered to.

## Discussion

In South Africa as in other settings university students comprise a large population of young people, but there is relatively little research focusing on the cultural factors influencing sexual risk-taking behaviour especially among Black male students in institutions of higher learning [43–45]. The assessment of narrated forms of sexual risk behaviours and contextual factors are a vital step toward designing effective strategies and interventions [44]. This is particularly important because students are potential role models and future leaders in an increasingly changing society. There is a need to recognize the role of cultural influences on risk-taking sexual practices that pose a serious health concern for the student population [46].

[47] Our findings support the factthat it is through socialisation that an individual learns about acceptable and unacceptable norms, which include norms of sexual behaviour [32, 48].

Sexual behaviour among male students to a large extent is influenced by society, immediate family, communities and peers. These findings are in line with the sexual script theory that states that sexuality is shaped through experiences, and meanings are developed through social encounters within a historical period [47, 49]. The values taught in the family, the interaction of these young men within the social-context of the university, and the infusion of different social and cultural backgrounds cannot be disregarded.

In a context that exerts pressure on young men to be sexually active, religious family background did not provide significant moral frameworks in influencing and shaping the sexual decisions of young men. This coincides with the study by Van Staden and Badenhorst (50), who reported that many students become sexually active as they move away from their homes and enter a developmental phase during which experimentation and risk-taking with a variety of sexual practices occur. Notably, but not surprising was the role played by peers in influencing the desire for some of the young men to engage in sexual activities. This finding concurs with a study conducted by [51] that reported on peer influence as a predisposing factor for risky sexual behaviour, particularly among men. The authors further argue that the role of peer influence should not be downplayed [51].

The role of fathers in educating young men about safer sex practices emerged from our findings, although such findings are contrary to dominant cultural norms. As in many African families, parents have been known to withhold important information about sexual activities and further promote messages of caution, fear and shame [52]. Findings from our study report the important role played by some fathers in educating their young men about safer sex practices. This warrants for more studies to be conducted on understanding the role that fathers can play in educating young boys at an early age about sexuality education, particularly among Black traditional families.

The study findings revealed the double standards existing in some cultures, where the dominant gender norms seemed to favour men more than women. For instance, the majority of the participants endorsed norms that allowed men more sexual freedom while prohibiting the same for women. This was not surprising as young women in the African culture are socialised to be subservient to men and accept male control over everything, including in sexual intimacy even to their detriment [5]. Gender norms continue to favour and protect men while further abusing and oppressing women especially in some African cultures [3].

Alcohol consumption was reported to play a vital role in facilitating sexual encounters for some participants in the study. The consumption of alcohol was revealed to contribute to risky sexual behaviours since participants reported on unprotected and inconsistent use of condoms when intoxicated. These findings are consistent with a study conducted by Chanakira et al. [45] that showed that alcohol was perceived as a social lubricant by most students, which often led to risky sexual behaviours. The findings further revealed that young men exchanged alcohol for sex with young women. This was troubling because of the reported sexual risk-taking which included non-condom use and random ‘hookups’ because of intoxication. Moreover, the findings further highlight the vulnerability of young women and what they can be subjected to when they accept such transactions. Some women have been sexually abused when drunk in university settings, however, they never report this because of fear, stigma and victimization [51].

## Conclusion

Although this study was undertaken at a single tertiary education facility in South Africa, the results are of concern and suggest that further studies need to be undertaken at other institutions, both within South Africa and in other countries where similar socio-cultural values may exist, in order to develop appropriate interventions. This study has some limitations. For example, under normal circumstances, participants may have fear in narrating their behaviours and conducts openly. Therefore, focus group discussions would have been much better if they were coupled with in-depth interviews. However, these were university student at a high academic level and were free to talk openly due to the liberal attitudes adopted within the space of the university. We do, however, recommend that future studies should utilise other alternative methods to minimise social desirability bias. The small sample size means the groups might not be a good representation of the larger population.

Our findings have been able to substantiate previous studies [21, 45, 53, 54] that young men’s sexual behaviours are influenced by different forms of norms that exist within their cultural backgrounds. These norms were revealed to shape the sexual behaviour, sexual interest and sexual beliefs of the participants in the study. It is also important to note that some of the young men in the study rejected the normalised risk-taking among men, and opted for alternative versions of being a man. This confirms that although social structures prescribe gender norms, however, individuals make gender a reality through their everyday behaviour, which can either support or undermine established gender structures. Finally, the misconceptions and lack of knowledge regarding safer sex practices among young people still seem to prevail and need even more urgent attention.

We thus recommend that health promotion programs at an institution of higher learning should enable young people to resist existing harmful expectations and further facilitate changing the expectations around them especially expectations that put young people’s health at risk. As such, unearthing the relationship between young people’s cultural norms and sexual behaviours is critical to intervention design tailored for this group of population. Furthermore, the results suggest the necessity of sex-education programmes for university students considering the low proportion of students entering university with basic sex education.

## Data Availability

The data supporting the findings of the article belongs to the University of KwaZulu-Natal and are currently being used for the Ph.D. of the lead author. This data will be shared on request from the lead author upon completion of her Ph.D.
